# Airway Thiol-NO Adducts as Determinants of Exhaled NO

**DOI:** 10.3390/antiox10101527

**Published:** 2021-09-26

**Authors:** Megan Pophal, Zachary W. Grimmett, Clara Chu, Seunghee Margevicius, Thomas Raffay, Kristie Ross, Anjum Jafri, Olivia Giddings, Jonathan S. Stamler, Benjamin Gaston, James D. Reynolds

**Affiliations:** 1 Institute for Transformative Molecular Medicine, Case Western Reserve University, Cleveland, OH 44106, USA; mpophal@nd.edu (M.P.); zxg198@case.edu (Z.W.G.); cxc653@case.edu (C.C.); jss156@case.edu (J.S.S.); jxr343@case.edu (J.D.R.); 2 Department of Population and Quantitative Health Sciences, Case Western Reserve University, Cleveland, OH 44106, USA; sxp2@case.edu; 3 Division of Pediatric Pulmonology, Department of Pediatrics, University Hospitals Cleveland Medical Center, Cleveland, OH 44106, USA; Thomas.Raffay@UHhospitals.org (T.R.); Kristie.Ross@UHhospitals.org (K.R.); anjum.jafri@case.edu (A.J.); Olivia.Giddings@UHhospitals.org (O.G.); 4 Division of Cardiology, Department of Medicine, University Hospitals Cleveland Medical Center, Cleveland, OH 44106, USA; 5 Harrington Discovery Institute, University Hospitals Cleveland Medical Center, Cleveland, OH 44106, USA; 6 Wells Center for Pediatric Research, Department of Pediatrics, Indiana University School of Medicine, Indianapolis, IN 46202, USA; 7 Department of Anesthesiology & Perioperative Medicine, University Hospitals Cleveland Medical Center, Cleveland, OH 44106, USA

**Keywords:** *S*-nitrosoglutathione, nitric oxide, asthma, GSNO reductase

## Abstract

Thiol-NO adducts such as *S*-nitrosoglutathione (GSNO) are endogenous bronchodilators in human airways. Decreased airway *S*-nitrosothiol concentrations are associated with asthma. Nitric oxide (NO), a breakdown product of GSNO, is measured in exhaled breath as a biomarker in asthma; an elevated fraction of expired NO (F_ENO_) is associated with asthmatic airway inflammation. We hypothesized that F_ENO_ could reflect airway *S*-nitrosothiol concentrations. To test this hypothesis, we first studied the relationship between mixed expired NO and airway *S*-nitrosothiols in patients endotracheally intubated for respiratory failure. The inverse (Lineweaver-Burke type) relationship suggested that expired NO could reflect the rate of pulmonary *S*-nitrosothiol breakdown. We thus studied NO evolution from the lungs of mice (GSNO reductase ^−/−^) unable reductively to catabolize GSNO. More NO was produced from GSNO in the ^−/−^ compared to wild type lungs. Finally, we formally tested the hypothesis that airway GSNO increases F_ENO_ using an inhalational challenge model in normal human subjects. F_ENO_ increased in all subjects tested, with a median t_1/2_ of 32.0 min. Taken together, these data demonstrate that F_ENO_ reports, at least in part, GSNO breakdown in the lungs. Unlike GSNO, NO is not present in the lungs in physiologically relevant concentrations. However, F_ENO_ following a GSNO challenge could be a non-invasive test for airway GSNO catabolism.

## 1. Introduction

Thiol-NO adducts such as *S*-nitrosoglutathione (GSNO) are endogenous bronchodilators present in normal human airways in concentrations near the IC_50_ for causing smooth muscle relaxation [1,2,3]. A reduction in GSNO levels is associated with respiratory failure in asthma [4]. GSNO is generated by NOS isoforms [5,6,7,8,9], other metalloproteins [10], and by acidic conditions, particularly in the presence of mM glutathione concentrations found in the distal airway [11]. GSNO can be broken down homolytically to form oxidized glutathione and NO [12,13,14], but it is also catabolized to hydroxylamine, ammonia and other reduction products by GSNO reductase (GSNOR) [15,16], *S*-nitroso Coenzyme A reductase (ScoR/Akr1a1) [17], and other enzymes such as carbonyl reductase.

The fraction of exhaled NO (F_ENO_) is often used to identify the degree of airway inflammation in children [18,19] and adults [20] with asthma. As a measure of inflammation, it has been assumed that elevations in F_ENO_ reflect an increase in NO production by inducible NO synthase (iNOS, or NOS2) [21,22,23]. However, the physiological reality is not that simple. For example, F_ENO_ is strongly affected by airway pH [24,25], the pulmonary microbiome, [26], and oxidative stress [5]. Of note, human lung concentrations of free NO (nM range)—unlike those of GSNO—are not sufficient to cause smooth muscle relaxation [27,28]. We therefore hypothesized that F_ENO_ measurements could be valuable as assays to report the presence and metabolism of airway GSNO and began with a small pilot study in intubated children with adult respiratory distress syndrome (ARDS), followed much later by two confirmatory studies, one in a murine model system and one in adult human volunteers.

## 2. Materials and Methods

### 2.1. Regulatory Information

#### 2.1.1. Human Subjects Research

The initial study in intubated patients with respiratory failure was a non-interventional study that investigated the relationship between F_ENO_ and airway *S*-nitrosothiols (SNOs), chiefly GSNO. It was conducted in the Pediatric Intensive Care Unit (PICU) at the Naval Medical Center in San Diego. The study was approved by the Clinical Investigation Department, Naval Medical Center, San Diego. Written informed consent was obtained from each child’s parent(s) or guardian(s) prior to their ward’s participation. The experimental measures were analyzed offline and were not used to guide clinical care. Registration of this observational study with a clinical trial database was not required; in fact, it was conducted before ClinicalTrials.gov (accessed on 20 May 2021) was developed as a repository. Note that these data were analyzed initially within a year after the study, and this analysis led to the hypothesis that GSNO may be broken down enzymatically in human lungs. However, these data were not published at the time. They were recently re-analyzed, and the result was the same. We consulted the Indiana University Center for Bioethics regarding whether or not it was appropriate to publish old data. Because the protocol was approved by the IRB at the time it conducted, their opinion was that it was appropriate to publish the data. Note also that a separate study had been published in 1993 in Boston [11], but did not include measures of mixed expired NO, and therefore did not identify the relationship discovered in the San Diego study.

Later, a clinical trial studying healthy adults involved assessing the responses to a single inhalational dose of GSNO. The work was approved by the Institutional Review Board of University Hospitals Cleveland Medical Center and conducted under FDA IND 137035. The trial was registered at clinicaltrials.gov (NCT03926741, accessed on 20 May 2021). UHCMC is an AAHRPP-accredited institution, and an independent data and safety monitoring board was in place to review the procedures and adjudicate any adverse events. Written informed consent was obtained from each individual prior to their voluntary participation in the study. All aspects of the study protocol followed good clinical practice guidelines.

#### 2.1.2. Animal Studies

The murine lung experiments were approved by the Institutional Animal Care and Use Committee of Case Western Reserve University; CWRU is an AAALAC-accredited and PHS-assured institution. Monitoring and husbandry of the mice complied with *The Guide for the Care and Use of Laboratory Animals*, 8th Edition (2011) and tissue procurement occurred following humane euthanasia was consistent with the AVMA Guidelines (2013).

### 2.2. Methods for Study 1

The relationship between exhaled NO and pulmonary SNO levels in children with respiratory distress.

The investigations began with a small pilot study that involved non-invasive tests. The patients had minimal pre-existing comorbidities (smoking, coronary artery disease, etc.), and the investigator with the hypothesis (B.G.) was a pediatric pulmonologist attending in the PICU. The study took place between July 1994 and June 1996 and involved intubated subjects with ARDS and/or pneumonia. Age, sex, and underlying diagnoses were recorded on intake sheets.

An aliquot of tracheal aspirate was obtained following routine undiluted suctioning from the endotracheal tube, performed for airway hygiene at the direction of the attending clinical team (i.e., no subject was suctioned for research purposes). Each sample was placed in a coded Eppendorf tube, frozen in liquid nitrogen, then stored for batch analysis. *S*-nitrosothiol (SNO) levels were subsequently quantitated using photolysis-chemiluminescence [11] by technical staff blinded to the patients’ conditions. At the same time as aspirate collection, exhaled breath was collected from a 2 m corrugated ventilator tube connected to the exhaust port of the ventilator (we previously determined, by identifying the point of steady-state CO_2_ mixing, that passage through at least 1.2 m of corrugated tubing was required to ensure complete gas mixing). This system provides mixed expired [NO], and similar methods are used for dead space calculations. The concentration of exhaled NO was then measured using a low range (0–1000 ppb) chemiluminescence NO analyser (Model 42, Thermo Environmental; Franklin, MA). For comparison with the SNO levels, mixed expired NO concentrations were expressed in nM (in gas phase) rather than ppb.

### 2.3. Methods for Study 2

In vitro generation of NO gas from GSNO.

To confirm that GSNO breakdown was a determinant of F_ENO_ evolved from the lungs, we next employed a mouse model. Lung homogenates were prepared fresh from wild type mice and mice lacking the GSNO reductase enzyme (GSNOR^−/−^). For wild type and GSNOR^−/−^ mice, 20 mg (100 uL) aliquots of homogenate from individual mice were placed in 15 mL chromatography vials containing 3 mL of phosphate buffered saline, supplemented with 300 uM NADH, 2 mM GSH, and 100 uM GSNO; control samples contained 100 uL of phosphate buffered saline (PBS), 300 uM NADH, 2 mM GSH, and 100 uM GSNO. Each vial was sealed with a rubber septum, and then incubated in a 37 °C water bath. Head space gas samples were withdrawn every 2 min using a gas-tight Hamilton syringe, then immediately injected into a Sievers Nitric Oxide Analyzer 280i (NO-chemiluminescence; Zysense, Weddington, NC, USA). The resultant NO signal from the analyzer’s photomultiplier tube was recorded in mVolts.

### 2.4. Methods for Study 3

Finally, to “close the loop”, we measured whether F_ENO_ levels in healthy human volunteers would increase following inhalation of GSNO. The inclusion criteria were broadly defined as healthy adults between the ages of 18 and 50 years of age with no pre-existing cardiopulmonary disorder or disease state that would impact oxygen exchange (e.g., asthma). The exclusion criteria included pregnancy, chronic medication use, active smokers/vapers or a history positive for use of tobacco products, or the presence of any acute or chronic disease. After providing written informed consent, pulmonary function testing (FVC and FEV_1_) was performed to confirm normal lung dynamics and general vital signs were obtained (heart and respiration rates, blood pressure, and pulse oximetry arterial oxygen saturation).

Drug challenge consisted of inhalation of 2.5 mL of a nebulized solution of 10 mM GSNO (GSNO was synthesized for our use under GMP conditions by Olon-Ricerca; Mentor, OH, USA). Vital signs were obtained at discrete intervals while spirometry testing was repeated 60 min after GSNO exposure. For the primary endpoint, F_ENO_ was quantified using a Niox Mino (Circassia Group PLC; Morrisville, NC, USA) before and at 10 min intervals up to 1 h after GSNO inhalation. Measurements were obtained by trained users and followed the manufacturer’s guidelines. As a final safety assessment, subjects returned 2 days post-exposure for another spirometry test. Note that we have previously used similar protocols to show that placebo diluent inhalation does not change F_ENO_ in humans [29,30].

### 2.5. Statistical Analysis

Data are presented as means ± standard deviation.

To compare the NO headspace signal between control, GSNOR^−/−^ and WT lung homogenate at 12 time points (ranging from 1 to 31 min), a repeated measure linear mixed model with heterogeneous first-order autoregressive covariance structure was used. The least square means of nitric oxide signal at each time point were obtained from the model with 95% confidence intervals. Pairwise comparisons among the three groups (wild type vs. control, wild type vs. GSNOR^−/−^, and GSNOR^−/−^ vs. control) were performed at each time point. After testing the pairwise comparisons, the p-values from the pairwise comparisons, using Hochberg’s step-up procedure for controlling the Family-wise error rate related to multiple tests, were examined.

In the healthy human F_ENO_ response to inhaled GSNO data, patient baseline characteristics were summarized as mean and standard deviation (SD) for continuous variables and frequency (percentage) for categorical variables. To examine the effect of inhaled GSNO on vital signs, blood pressure, and heart rate over time, a repeated measures linear mixed model with compound symmetric structure was used. FVC and FEV1 were measured at pre/post-GSNO inhalation which was measured by 60 min. Using a paired *t*-test, we examined the mean difference (change) in FEV1 and FVC pre- and post-GSNO inhalation.

To explore changes in F_ENO_ after GSNO exposure over time, a repeated measure linear mixed model with first-order autoregressive structure (adjusting for the baseline F_ENO_ measurement) was used. Pairwise comparisons between baseline and each time point were performed. After testing the pairwise comparisons, the *p*-values from the pairwise comparisons using Hochberg’s step-up procedure for controlling the Family-wise error rate related to multiple tests were examined. Association between F_ENO_ and BMI was tested using the mixed model adjusting for the baseline F_ENO_. Using the trapezoidal rule, the area under the curve (AUC) was calculated for each of the healthy subjects, noting that in our two previous studies, there was no increase in F_ENO_ with inhalation of diluent, so that there was no area under the curve [29,30]. To examine the effect of inhaled GSNO on vital signs (heart rate, blood pressure, % oxygenation) over time, a repeated measures linear mixed model with first-order autoregressive structure with adjusting for the baseline measure was used. Pairwise comparisons between baseline and each time point were performed. After testing the pairwise comparisons, the *p*-values from the pairwise comparisons using Hochberg’s step-up procedure for controlling the Family-wise error rate related to multiple tests were examined. FVC and FEV_1_ were measured pre- and post- GSNO inhalation, which was measured at 60 min. Using a paired *t*-test or Wilcoxon sign rank test, depending on the data distribution, the changes in FVC and FEV_1_ pre- and post- GSNO inhalation were examined. All tests were two-sided and adjusted *p*-values from the Hochberg method less than 0.05 were considered as statistically significant. Data were analyzed using SAS software version 14.1 (Cary, NC, USA).

## 3. Results

### 3.1. Study 1. Data from Children with Respiratory Failure

**Figure 1:**
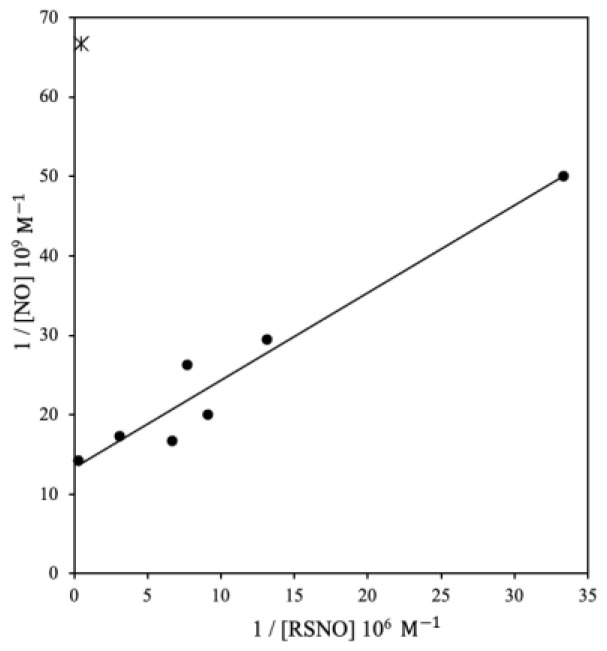
Inverses of SNO in tracheal aspirate vs NO levels collected in PICU. Measured by chemiluminescence, *S*-nitrosothiol concentrations in the tracheal aspirates were compared to the mixed expired [NO] found by the low-range chemiluminescence NO analyzer. The inverses of the data correlate with each other, as elevated SNO activity corresponds to high [NO]. The outlier in the top left represents a patient who suffered a pulmonary hemorrhage, with resulting (and expected) very low levels of NO and high levels of SNO.

The eight subjects enrolled in study 1 ranged from 5 months to 12 years of age; their demographic data are presented in Table 1. Post-hoc analysis revealed a strong positive correlation between mixed exhaled NO concentrations and tracheal aspirate SNO concentrations, and multiple curve fit analysis was most consistent with a relationship of inverses (Figure 1). In other words, NO evolution is high when *S*-nitrosothiol levels are elevated. The one outlier is a child who experienced pulmonary hemorrhage. In this subject, NO levels were very low and SNO levels very high, as would be expected due to the metabolism of SNOs by extravascular hemoglobin [6].

The positive correlation displayed in Figure 1 suggested to us that the mixed expired [NO] could be related to the rate of GSNO catabolism (possibilities studied in the second and third studies reported here): if this were the case, the graph could be considered in vivo as a Lineweaver-Burk plot to calculate enzymatic activity. By analogy, a Michaelis constant (K_M_) of 13 μM was found by extending the trendline in the figure to the *x*-axis. Our data demonstrate that the theoretical K_M_ (13 μM) is similar to the K_M_ for GSNOR (27 μM) [16]; i.e., the K_M_ of the in vivo Lineweaver-Burk plot detailing SNO catabolism corresponds to the in vitro K_M_ of the GSNOR enzyme. This relationship would require that the rate of GSNO catabolism would be reflected by mixed expired NO concentration. Therefore, we conducted a follow-up study in vitro in mice with and without GSNOR.

### 3.2. Study 2. Headspace Nitric Oxide Assay in Murine Lungs

**Figure 2:**
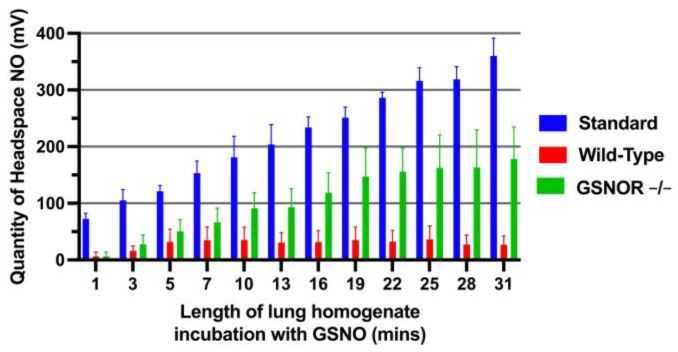
Mouse Lung Headspace NO. Murine lung homogenate samples from wild type and GSNOR^−/−^ mice as well as control samples were analyzed. The standard controls had the greatest quantity of headspace NO, followed by GSNOR^−/−^ mice and wild type. The absence of GSNOR results in decreased GSNO catabolism and therefore greater NO production.

The time course of evolved NO concentrations (expressed as means ± SD) for the three conditions (control and both wild type and GSNOR^−/−^ lung homogenates) are presented in Figure 2. The control samples (PBS) incubated with GSNO produced the highest averaged quantity of headspace NO, reported as the photomultiplier signal from the nitric oxide analyzer, averaging 217 ± 21.4 mV over 31 min. This reflects the inorganic breakdown of GSNO to NO. The GSNOR^−/−^ mice produced more headspace NO relative to PBS control, averaging 105 ± 36.5 mV, whereas the wild type samples averaged only 28.6 ± 18.5 mV (*p* < 0.0001).

Using pairwise comparisons between each of the three groups (*n* = 3 in each), we determined these group values were significantly different (*p* < 0.0001) and there were significant correlations between time and groups (*p* < 0.0001). The quantities of evolved NO between GSNOR^−/−^ vs. control and wild type vs. control differed significantly at all 12 time points, while after the 10 min of incubation, NO values for GSNOR^−/−^ vs. wild type lung tissue were also significantly different. With reduced (GSNOR^−/−^) or no (control) GSNO catabolism, significant quantities of headspace NO were produced, whereas in the presence of GSNOR (WT lung homogenate), GSNO was unable to produce NO (Figure 2).

### 3.3. Study 3. Healthy Human F_ENO_ Response to Inhaled GSNO

**Figure 3:**
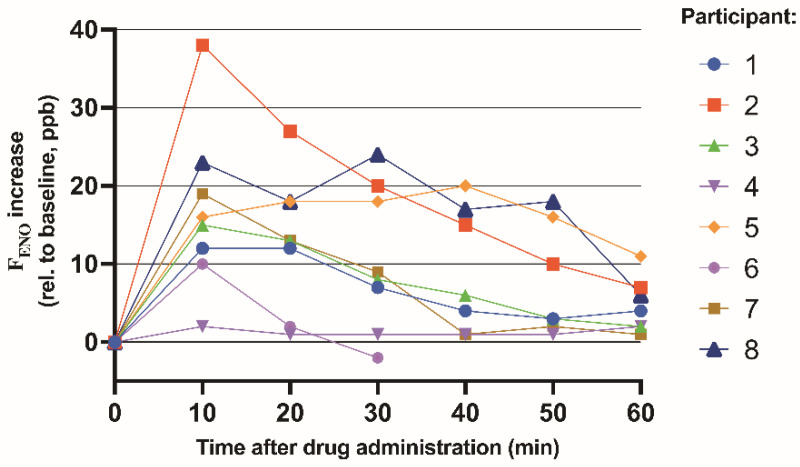
Effect of GSNO Inhalation on F_ENO_ Measurements. Initial F_ENO_ measurements were set at zero then F_ENO_ measurements after GSNO administration are depicted as the increase from the initial baseline.

**Figure 4:**
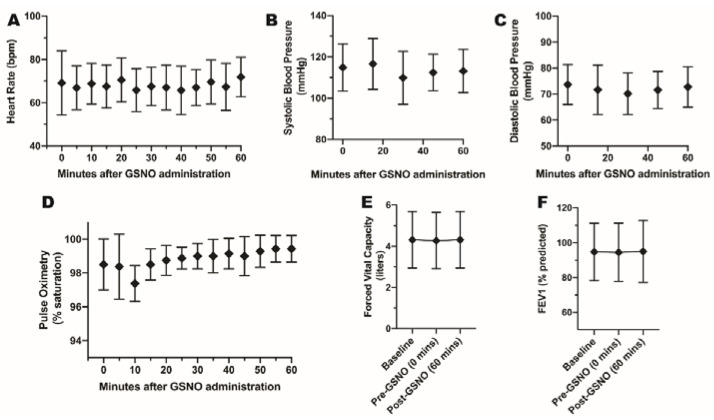
Physiologic Changes Following GSNO Administration. Heart rate (**A**), systolic blood pressure (**B**), diastolic blood pressure (**C**), pulse oximetry (**D**), FVC (**E**), and FEV1 (**F**) levels were recorded at intervals of 5 min (**A**,**D**), 15 min (**B**,**C**) or baseline, pre- and post-GSNO administration (**E**,**F**). No vital signs (**A**–**F**) reported here underwent a significant change after drug administration, suggesting that GSNO drug is safe.

We enrolled 8 non-asthmatic healthy adults (6/2; F/M) to study an in vivo physiologic response to inhaled GSNO (Figure 3); individual data for these volunteers are presented in Table 2. Following GSNO inhalation, there was little change in the monitored vital signs (Figure 4), consistent with findings from a previous exposure study conducted in cystic fibrosis patients [29]. Spirometry results before and after treatment were similar and were also unchanged at the 2-day post-exposure follow-up visit. In addition, no drug-related adverse effects were observed, supporting the general safety of GSNO administration in humans [29].

Inhalation of GSNO increased F_ENO_ in all subjects (Figure 3 and Table 2). Note that Participant 6 withdrew because the COVID-19 pandemic closed the study laboratory, and the subject subsequently left the area. Area under the curve (AUC) and t_1/2_ were calculated to define the F_ENO_ response. The group AUC mean was 573 ± 411 ppb*min^−1^, with a median of 452 ppb*min^−1^. The group mean t_1/2_ was 32.0 ± 13.5 min, with a median of 32.0 min and range of 44.0 min. These calculated parameters demonstrate an increase in F_ENO_ values for healthy individuals following a single test dose of GSNO, lasting at least 60 min after GSNO administration.

AUC and t_1/2_ values were determined for each subject and presented in Table 3. Qualitative secondary analysis identified two subsets of responders, low and high F_ENO_ generators. In the low cohort, participants 1, 3, 4, 6, and 7 had the lowest AUC values (Table 3) averaging 297 ± 191 ppb*min^−1^. In contrast, the high F_ENO_–generating cohort averaged a three-fold increase in AUC (1033 ± 100.0 ppb*min^−1^) and a four-fold increase in linear regression slopes (3.52 ± 0.16 ppb*min^−1^).

## 4. Discussion

Here, we have found that pulmonary GSNO breakdown is a determinant of pulmonary NO concentration, including F_ENO_. In study 1, [SNO] was a determinant of mixed expired NO in subjects with ARDS. In study 2, headspace NO evolved from murine lung homogenates was elevated when lungs were unable reductively to catabolize GSNO to ammonia (as opposed to NO). In study 3, inhalation of exogenous GSNO was shown to increase F_ENO_ in humans in vivo. Elevations in F_ENO_ are classically interpreted as simply reflecting increased iNOS activity associated with lung inflammation [18]. However, this is not a clear-cut relationship. For example, patients with severe airway inflammation, such as those with primary ciliary dyskinesia and cystic fibrosis [31,32], have low F_ENO_ levels, and administration of iNOS inhibitors to reduce lung inflammation have produced equivocal results. Additionally, NOS activity results in formation of nitrogen oxides other than NO that are both beneficial (GSNO) and toxic (peroxynitrous acid) [5]; and murine data are contradictory regarding the role of various NOS isoforms [33,34]. In the current work, we provide three lines of evidence that a major contributor to F_ENO_ is the status of *S*-nitrosothiol metabolism in the lungs.

GSNO is a major member of the SNO class of endogenous signaling molecules in the lungs [5]. In human airways, it is a significant airway smooth muscle relaxant. It is formed both in acidic environments in the lungs and elsewhere, as well as by metalloproteins such as NOSs [11,26,29]. It is catabolized [12,13,14], and its activity is regulated, by GSNO reductase (GSNOR), SNO-CoA reductase (SCoR) [17], and other enzymes [35].

The relationship between GSNOR and headspace NO was analyzed in murine lung homogenates (study 2). The reductive catabolism of GSNO by GSNOR decreased the headspace NO concentration, reflecting evolution of NO from GSNO, in WT vs. GSNOR^−/−^ mouse lung tissue. GSNOR^−/−^ knockout mice yielded greater quantities of headspace NO compared to mice expressing wild-type GSNOR (Figure 2). GSNOR breaks down GSNO to hydroxylamine and ammonia, preventing the homolytic formation of NO from GSNO [36]. It should be noted that hydroxylamine can, in some circumstances, be oxidized to NO. However, we do not believe this to occur appreciably in our assay due to the minimal level of NO evolved from murine lung homogenate expressing wild-type GSNOR. The differences seen in Figure 2 are consistent with the understanding that with no (control) or reduced (GSNOR^−/−^) enzymatic catabolism, GSNO ceases to break down to ammonia and is therefore able to form NO. By extension, these data also support the concept that the level of GSNO in murine lung tissue significantly impacts the evolution of NO from the lungs. In the absence of GSNO reductase, GSNO in the knockout mice was not reduced and therefore could form greater quantities of headspace NO (Figure 2), particularly after the 10 min timepoint. Our findings support the notion that GSNO breakdown is a determinant of NO evolved from murine lungs.

In the initial study, a significant relationship was identified when comparing the inverse of the mixed expired [NO] and inverse of [GSNO] among children with respiratory failure, in a relationship reminiscent of Michaelis–Menten kinetics, assuming that [NO] reflects the rate of enzymatic GSNO breakdown (Figure 1). This relationship is what originally led to the hypothesis that there could be enzymatic GSNO breakdown in the lungs. This hypothesis later proved to be correct and led ultimately to the creation of the GSNOR ^−/−^ mouse. The observed correlation supports the concept that in children with respiratory failure, airway SNO catabolism can be a major determinant of mixed expired NO concentration, as it appeared to be catabolism rather than anabolism that drove the NO-SNO relationship. Thus, these pediatric data initially led us to suspect that NO evolution in the lung reflected *S*-nitrosothiol catabolism. It should be noted that the outlier, a patient with pulmonary hemorrhage, is well explained when considering known hemoglobin *S*-nitrosothiol synthase function [6,37]. It is important to emphasize that the mixed expired NO measure is a collection method that is different from online, flow dependent F_ENO_ measures commonly made in clinic; but it provides complementary information. Of note, it will be of interest in the future to compare mixed expired [NO] to dead space/CO_2_ measurements.

With correlations identified between GSNO and NO in mice and in respiratory failure patients, non-asthmatic healthy human volunteers were tested to determine a healthy F_ENO_ response to inhaled GSNO. During the GSNO challenge test, inhaled GSNO increased the F_ENO_ values in healthy subjects. Note that inhalation of the diluent placebo alone does not increase F_ENO_ in humans [29,30], and that repeated F_ENO_ maneuvers do not increase F_ENO_ values [38]. Thus, airway GSNO can be a determinant of F_ENO_ in healthy humans as well as in those with respiratory failure. The results suggest that, in this case, F_ENO_ levels are dependent on homolytic GSNO breakdown, rather than simply reflecting increased airway inflammation. We hypothesized that with increased GSNO reductase activity, less NO would be evolved [5]. There were two subsets of participants differing by AUC and slopes of linear regression. This finding suggests that even within a relatively homogenous population, there is phenotypic variation in GSNO metabolism in the lungs. Future studies may provide further insight regarding GSNO catabolic enzyme activity in certain populations. Thus, the GSNO challenge in the lung function lab could be used as a test for GSNO metabolism. This study also confirms that GSNO is safe and well-tolerated [29].

The study has some limitations, including relatively low numbers of subjects. Both human trials (study 1 and study 3) were designed as pilot investigations, and larger future trials will help to confirm our findings.

## 5. Conclusions

These studies demonstrate that pulmonary GSNO affects F_ENO_. This relationship between GSNO and NO has important implications. F_ENO_ levels are not always useful as diagnostic tests for airway inflammation [3,6], but could be employed as readouts in challenge tests in the lung function lab that are designed to evaluate airway pH [25,37], the airway microbiome [26], and GSNO metabolic status [31,32]. GSNO metabolism, in turn, is increasingly recognized as important to asthma pathophysiology [5,15,39].

## Figures and Tables

**Table 1:** Intensive Care Unit (ICU) subject characteristics at study entry.

Pediatric Participant	Age	Sex	Underlying Diagnosis/es	[NO] (nM)	[SNO] (uM)
1	2 y/o	Female	Urosepsis	0.07	3.4
2	6 m/o	Male	Enterococcal sepsis	0.05	0.11
3	3 y/o	Male	Aspiration pneumonia	0.058	0.32
4	9 m/o	Male	*Pneumocystis jirovecii* pneumonia	0.06	0.15
5	12 y/o	Female	*Pseudomonas aeruginosa* sepsis	0.015	2.1
6	5 m/o	Female	Pseudomembranous colitis, *C. difficile*, *Klebsiella* sepsis	0.02	0.03
7	5 y/o	Female	*Candida* sepsis, Acute Lymphoblastic leukaemia), Pulmonary hemorrhage	0.034	0.076
8	1 y/o	Male	Community acquired pneumonia/ARDS	0.038	0.13

Between July 1994 and June 1996, subjects with ARDS and/or pneumonia were enrolled (protocol *S*-94-LH0000-016). Subject characteristics including age at enrollment, sex and underlying diagnosis are presented in this table.

**Table 2:** Participants’ characteristics and baseline and maximum post-GSNO F_ENO_ measurements (Study 3).

Adult Participant	Gender	Age (Years)	Smoking Status	Passive Smoke Exposure during Childhood	Current Exposure to Environmental Smoke	Baseline Pulse Oximetry (% sat)	Baseline F_ENO_ (ppb)	Peak F_ENO_ (Post- GSNO, ppb)
1	Female	26	Non-smoker	No	None	98	16	28
2	Female	27	Non-smoker	Yes	None	99	5	43
3	Female	19	Non-smoker	No	None	97	5	20
4	Female	19	Non-smoker	Yes	None	100	17	19
5	Female	21	Non-smoker	No	None	100	11	31
6	Female	26	Non-smoker	No	None	96	50	60
7	Male	23	Non-smoker	No	None	98	19	38
8	Male	19	Non-smoker	No	None	100	59	83

Participating subjects in study 3, 8 non-asthmatic healthy adults, were enrolled following specific inclusion criteria. Their demographic information, including gender, age, BMI, race, household income, heart rate and pulse oximetry, are presented above. Participants performed the GSNO Challenge Test after obtaining proper informed consent.

**Table 3:** Area Under the Curve and t_1/2_ of F_ENO_ Measurements.

Participant	AUC (ppb*min^−1^)	t_1/2_ (min)
1	400	34
2	1135	32
3	460	33
4	70	20
5	935	>60
6	110	16
7	445	29
8	1030	58

Area under the curve calculations using trapezoidal rule on the healthy adult human volunteer F_ENO_ curves from Figure 3. AUC = Area Under the Curve, given in units of ppb*min^−1^ = ppb per minute.

## Data Availability

Data is contained within the article; and are also available upon request.
